# Reaching out for hope - a peer support program

**DOI:** 10.1186/2050-2974-2-S1-O63

**Published:** 2014-11-24

**Authors:** Julie Purcell, Sue Lister, Julie McCormack, Jemma Caswell, Kathy Logie, Stephanie Wade, Mandy Stringer

**Affiliations:** 1 Eating Disorders Program, Specialised CAMHS, Child and Adolescent Health Service, Princess Margaret Hospital, Perth, Australia; 2 Body Esteem Program, Women's Healthworks, Perth, Australia

## 

The value and need for consumer involvement in the delivery of mental health services is becoming increasingly recognised and acknowledged both throughout government and non-government organisations. The aim of consumer and carer involvement and engagement is, ultimately, to improve the quality of health care provided.

The Child and Adolescent Mental Health Service (CAMHS) Eating Disorders Program (EDP), is a specialised service located at Princess Margaret Hospital. CAMHS has always had a strong investment in consumer focus and participation and have consistently included consumer input in to the current clinical services. In 2013, funding was obtained from a Capacity Building Grant which allowed for collaboration between CAMHS EDP and Women’s Healthworks (WHW), Body Esteem Program (BEP). From this collaboration a peer support program called “Reaching out for Hope,” was piloted. The “Reaching out for Hope” program was designed to allow young people attending the PMH EDP service to hear the experiences of others who once had an eating disorder, but now manage life without one. Delivery of the group program is based on peer support workers having a reciprocal and equal, non-clinical, non-expert relationship with participants.

Developing this program has involved several steps including ethics and funding approval, integration of research, clinical services and consumer input. This presentation will detail the program from inception to delivery, including details from the final report focusing on identified strengths and limitations. The presentation will be delivered collaboratively by CAMHS EDP and WHW BEP staff.

This abstract was presented in the **Peer Support** stream of the 2014 ANZAED Conference.

